# Authors’ Reply: Enhancing AI-Driven Medical Translations: Considerations for Language Concordance

**DOI:** 10.2196/71721

**Published:** 2025-04-11

**Authors:** Joyce Teng, Roberto Andres Novoa, Maria Alexandrovna Aleshin, Jenna Lester, Kira Seiger, Fiatsogbe Dzuali, Roxana Daneshjou

**Affiliations:** 1 Department of Dermatology, Stanford University, 700 Welch Rd, Stanford, 94305, United States, 1 6507236493; 2 Department of Pathology, Stanford University, Redwood City, CA, United States; 3 Department of Dermatology, University of California, San Francisco, CA, United States; 4 Department of Dermatology, University of Washington, Seattle, WA, United States; 5 Department of Biomedical Data Science, Stanford University, Stanford, CA, United States

**Keywords:** ChatGPT, artificial intelligence, language, translation, health care disparity, natural language model, survey, patient education, accessibility, preference, human language, communication, language-concordant care

We appreciate the thoughtful insights shared by Quon and Zhou [1] regarding our study on the application of ChatGPT in translating patient education materials [2]. We wholly agree that the linguistically distinct languages, such as Mandarin, can present challenges in capturing all the nuances and achieving precise translations.

In response to the comment regarding the use of multiple prompts, we acknowledge the complexity and variability in artificial intelligence (AI)-generated translations. However, it is important to consider the practical limitations within a clinical setting. Asking providers to use various prompts in real time may not be feasible due to time constraints and the need for efficiency in patient care. We believe that focusing on a single, effective prompt can streamline the translation process while we explore avenues for improvement in the AI’s capabilities. This could be a productive avenue for future research.

Addressing the concern regarding the reliance on board-certified dermatologists for post-translation review, we want to clarify that, in addition to being board-certified dermatologists, all reviewers were native speakers in the language they reviewed, including fluency in Mandarin at a college level. This proficiency allows for a confluence of both clinical and linguistic insights when evaluating translations, reinforcing the validity of our findings. We appreciate the importance of rigor in translation review and remain committed to enhancing the integrity of our translated materials.

Overall, while we recognize the areas where ChatGPT can improve, we also see its current utility as a valuable tool for expanding access to language-concordant care in clinical settings. Our study serves as a helpful step toward identifying and addressing the limitations of AI translations, and we welcome continued dialogue to refine these practices.

## Abbreviations

AI: artificial intelligence
